# Supplementary material for: The Inhibitory Role of Rab11b in Osteoclastogenesis through Triggering Lysosome-Induced Degradation of c-Fms and RANK Surface Receptors
Source: Int J Mol Sci. 2020 Dec 8;21(24):9352. doi: 10.3390/ijms21249352 (PMC7763820; doi:10.3390/ijms21249352)
Supplement: Supplementary file 1 [file ijms-21-09352-s001.pdf]

International Journal of
Molecular Sciences

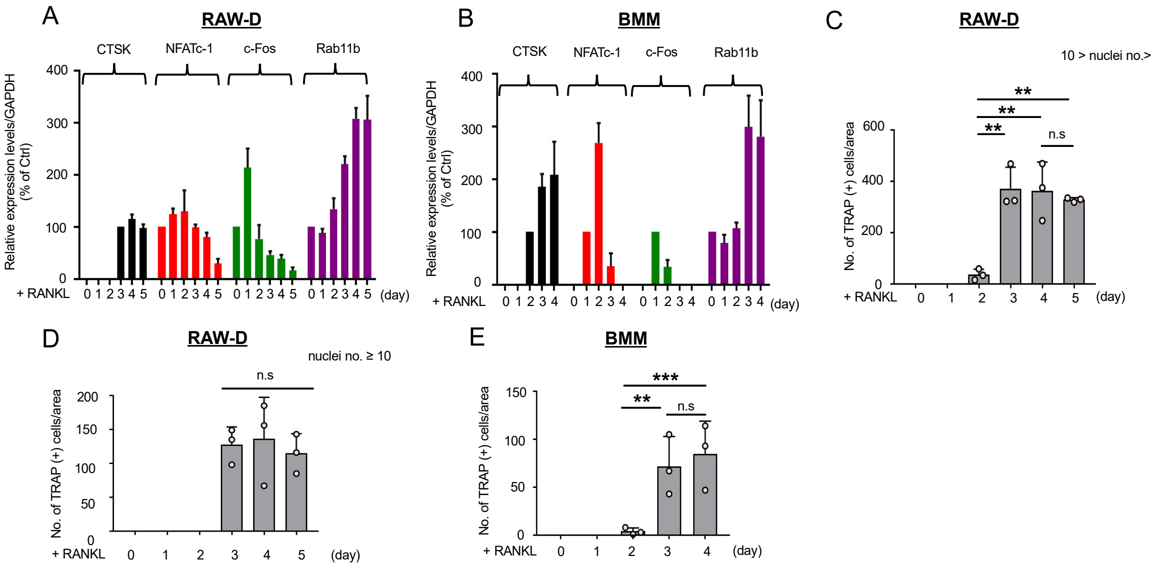

Figure S1. (**A**, **B**) The densitometry qualification of bands illustrating endogenous levels of CTSK, c-Fos, NFATc1, Rab11b was shown in RAW-D cells (**A**, related to Figure 1B) or BMMs (**B**, related to Figure 1D) over a time course of RANKL (300 ng/mL) stimulation. To c-Fos, NFATc-1, Rab11b, bands (at day 2, 3, 4, or 5) were calculated as a percentage of that of day 0 thereof whereas those of CTSK (day 4, or day 5) were evaluated as a percentage of that of day 3 thereof. The shown data were representative of three independent repeats. (**C**, **D**) The number of TRAP-positive multinucleated osteoclasts derived from RAW-D cells harboring nuclei number in a range of 3 to 10 nuclei (**C**) or more than 10 nuclei (**D**) per viewing field was counted (related to Figure 1E). Mean ± SD of three independent repeats. **$P< 0.01$; ns, nonsignificant (Student's *t* test). (**E**) The number of TRAP-positive multinucleated osteoclasts derived from BMMs harboring nuclei number more than 3 per viewing field was counted (related to Figure 1F). Mean ± SD of three independent repeats. **$P< 0.01$; ***$P< 0.001$; n.s, nonsignificant (Student's *t*-test).

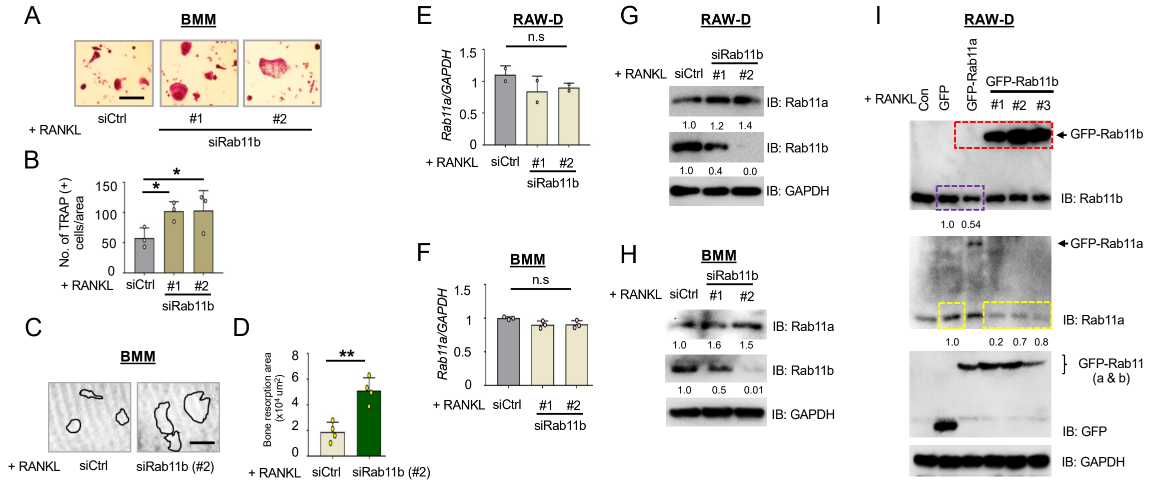

Figure S2. (**A**) BMMs were transfected with either the non-targeting siRNA (siCtrl) or one of two different types of Rab11b-specific siRNA (Rab11b siRNA #1 or #2). After 3 days of RANKL (300 ng/mL) stimulation, TRAP-staining was carried out. The images of osteoclasts were photographed by the Olympus FSX100 microscope. The shown images were representative of three independent repeats. *Bars* 200μm. (**B**) The number of TRAP-positive osteoclasts harboring 3 or more nuclei per viewing field was counted. Mean ± SD of three independent repeats; **P*< 0.1 (Student's *t* test). (**C**) RAW-D cells were transfected with either the non-targeting siRNA (siCtrl) or Rab11b-specific siRNA (siRab11b #2). After treated with M-CSF (30 ng/mL) and RANKL (500 ng/mL) for 7-10 days, the bone-resorption areas were photographed sing the Floid cell imaging station. The shown images were representative of three independent repeats. *Bars* 100μm. (**D**) The size of bone-resorption areas was determined by ImageJ software. Mean ± SD of three independent repeats; **P*< 0.05 (Student's *t* test). (**E**, **F**) RAW-D cells or BMMs were transfected with either the non-targeting siRNA (siCtrl) or one of two different types of Rab11b-specific siRNA (siRab11b #1 or #2). After 3 days of RANKL (300 ng/mL) treatment, total Rab11a mRNA was prepared, and analyzed by qRT-PCR in osteoclasts derived from RAW-D cells (**E**) or derived from BMMs (f). Mean ± SD of triplicate samples; **P*< 0.05 (Student's *t*-test). (**G**, **H**) The control- or Rab11b-silenced RAW-D cells (G) or BMMs (H) were pre-treated with RANKL (300 ng/mL) for 3 days, followed by WB analysis of Rab11a and Rab11b expression levels. The data shown were the representative of three independent repeats. (**I**) RAW-D cells (wild type) and RAW-D cells expressing GFP, GFP-Rab11a, or one of three different types of GFP-Rab11b were pre-treated with RANKL (300 ng/mL) for 3 days, followed by WB analysis of GFP-Rab11a, GFP-Rab11b, Rab11a, and Rab11b expression levels.GFP-Rab11b expression levels in the RAW-D cells expressing GFP-Rab11a and GFP-Rab11b were remarked by a red rectangle so as to confirm the specificity of the primary antibody of Rab11b used throughout this study. GAPDH was used as a loading control. The densitometry reading of Rab11b levels was measured underneath from second to third lanes remarked by a purple rectangle. The second lane was arbitrarily set as 1.0. The densitometry reading of Rab11a levels was measured underneath second lane and from the 4th to 6th lanes remarked by the yellow rectangles. The 2nd lane was arbitrarily set as 1.0. The blue asterisk indicated GFP-Rab11a expression. The data were the representatives of two independent repeats.

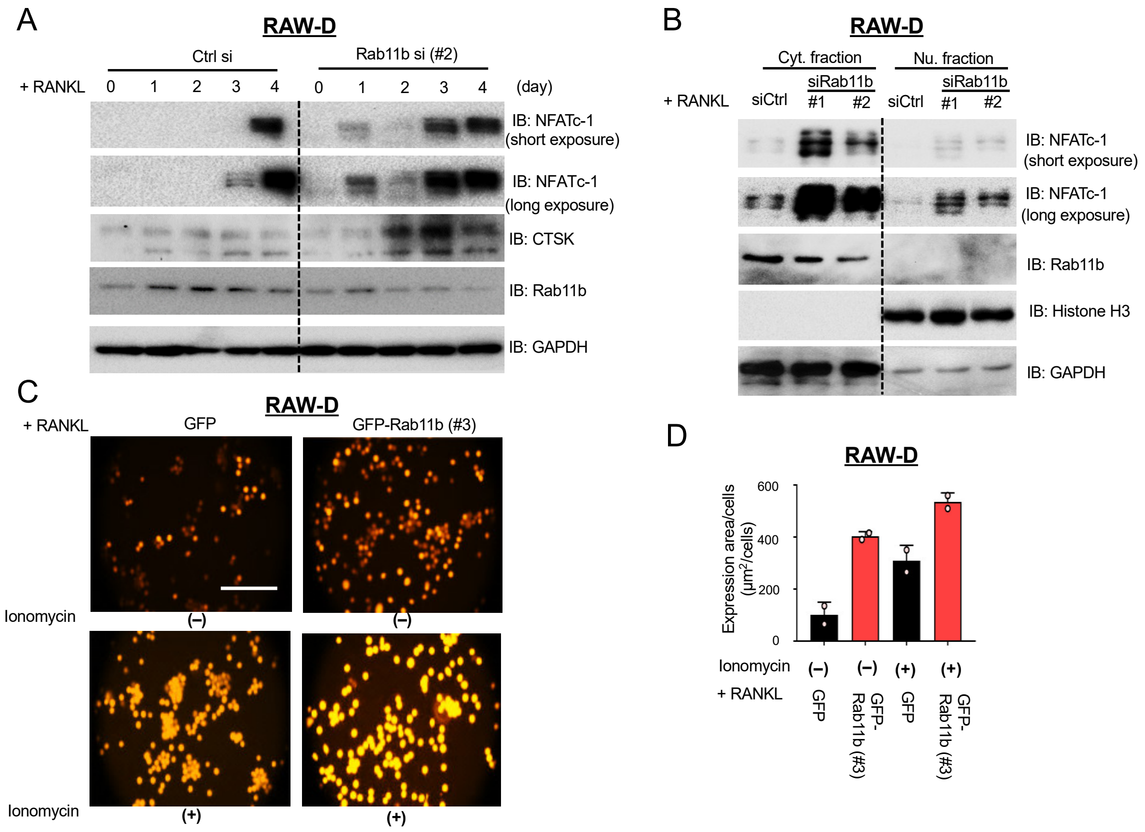

Figure S3. (**A**) RAW-D cells were transfected with non-targeting siRNA or Rab11b siRNA (#2). Cells were treated with RANKL (300 ng/mL) over a time course (5 days), followed by WB analysis of CTSK, NFATc-1 expression levels. GAPDH was used as a loading control. The shown data were the representative of two independent repeats. (**B**) Cytosolic and nuclear fractions were prepared from osteoclasts stimulated with RANKL (300 ng/mL) over 3 days, following the transfection of non-targeting siRNA or one of two different type of Rab11b siRNAs (#1 or #2) into RAW-D cells. The cytosolic and nuclear fractions were subjected to SDS-PAGE and WB analysis of NFATc-1 expression level. Histone H3 and GAPDH were used the nuclear and cytosolic markers, respectively. (**C**) The effects of ionomycin on [Ca^{2+}_i] oscillation in RAW-D cells expressing GFP or GFP-Rab11b (type #3), following 2 days of RANKL (300 ng/mL) treatment. Cells were loaded with 1 μM Cal-590 for 1 h in serum-free αMEM, washed, and treated with or without 10 μM ionomycin. The cells were analyzed by Olympus Uplsapo 10X. (**D**) The [Ca^{2+}_i] fluorescent intensities were measured and analyzed by Image J software. Mean ± SD of two independent repeats. *Bars* 100μm.

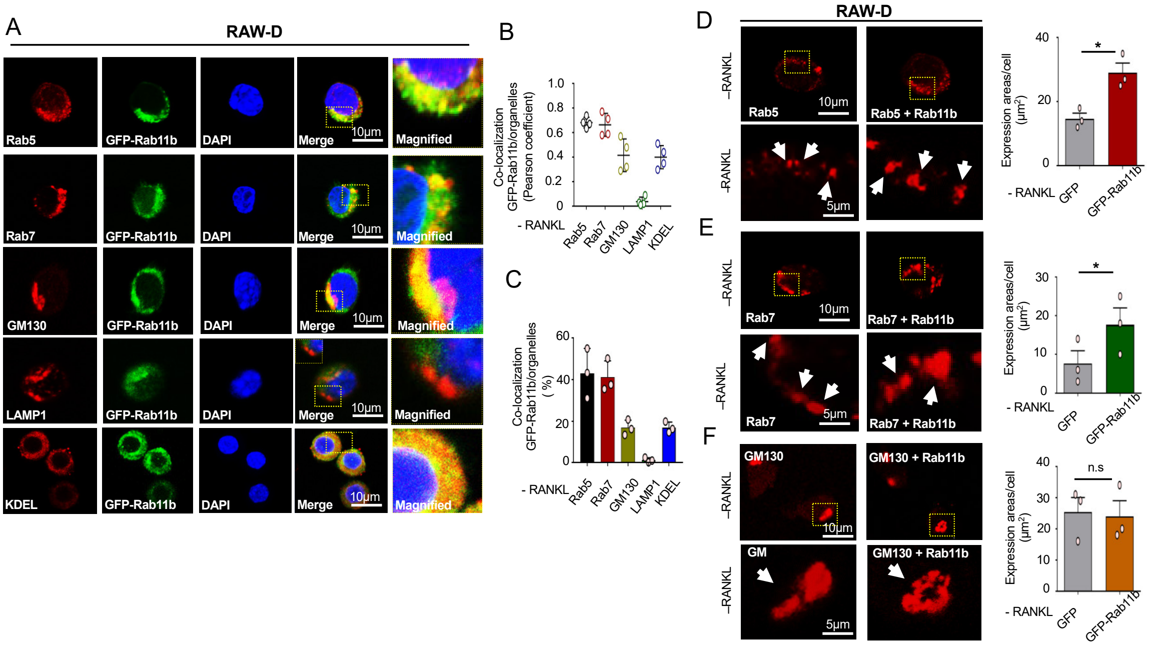

Figure S4. (**A**) RAW-D cells stably expressing GFP-Rab11b (green) were seeded on cover glasses with fixation and permeabilization of 0.2% Triton X-100 in PBS, and subsequently reacted with one of the antibodies against Rab5, Rab7, MG130, LAMP1, or KDEL (red, as indicated) that are specific markers for early endosomes, late endosome, Golgi complex, lysosomes, or endoplasmic reticulum, respectively. DNA was stained with DAPI (blue). Specific regions of interest within a field by yellow boxes are magnified on left side of each image it was taken from. The yellow color was referred as a merge between green and red colors. Mean ± SD of three independent repeats. Scale bar: 10 μm. (**B**, **C**) GFP-Rab11b co-localization with Rab5, Rab7, MG130, LAMP1 and KDEL was determined and evaluated by Pearson coefficient (**B**) or by color threshold analysis (**C**) using Fiji/ImageJ on at least 4 cells. **P*<0.05, ***P*<0.01, ****P*<0.001, n.s. nonsignificant (Student's *t*-test). (**D**, **E**, **F**) The osteoclasts derived from RAW-D cells stably expressing GFP or GFP-Rab11b were fixed and permeabilized with 0.2% Triton X-100, and stained with the specific antibodies to detect (d) Rab5 (red), (e) Rab7 (red), and (f) MG130 (red). The images were captured by a confocal laser microscopy. The particle size was measured by (μm^2) using ImageJ (shown in *right side*). **P*< 0.05, n.s. nonsignificant (Student's *t*-test).

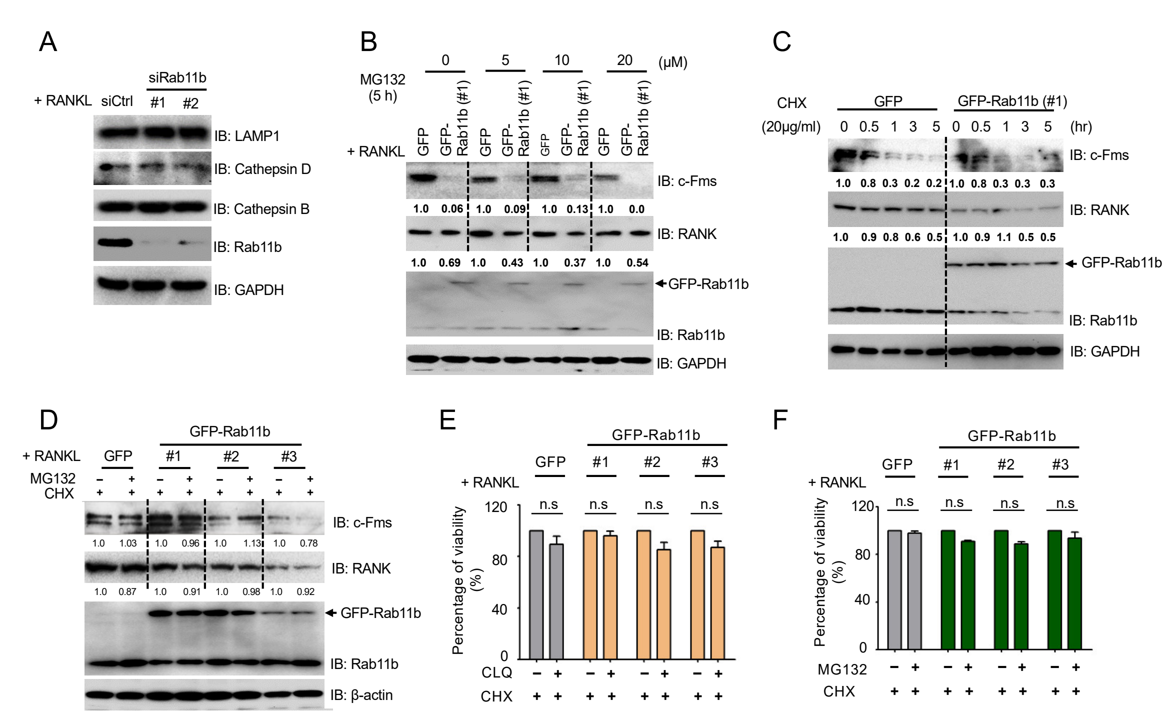

Figure S5. (**A**) RAW-D cells were transfected with non-targeting siRNA (siCtrl) or one of two different types of Rab11b siRNAs (#1 and #2), followed by RANKL (300 ng/mL) treatment for 3 days. The WB analysis of endogenous levels of LAMP1, a specific lysosomal receptor, and two lysosomal enzymes, Cathepsins B and D was done. GAPDH was used as a loading control. The shown data was the representative of two independent repeats. (**B**) RAW-D cells expressing GFP or GFP-Rab11b (type #1) was pre-treated with RANKL (300 ng/mL) for 3 days before incubated with MG132 (0, 5, 10, 20 μM) for 5 h. The WB analysis of endogenous levels of c-Fms, RANK, and Rab11b was done. GAPDH was used as a loading control. The shown data was the representative of two independent repeats. (**C**) RAW-D cells expressing GFP or GFP-Rab11b (type #1) were pre-treated with RANKL (300 ng/mL) for 3 days before incubated with CHX (20 μg/mL) over a time course (0, 0.5, 1, 3, 5 h). The WB analysis of endogenous levels of c-Fms, RANK and Rab11b was done. β-actin was used as a loading control. The shown data was the representative of two independent repeats. (**D**) RAW-D cells expressing GFP or one of three different types of GFP-Rab11b (type #1, #2, and #3) was pre-treated with RANKL (300 ng/mL) for 3 days before incubated with the combination of MG132 (10 μM) and CHX (20 μg/mL) for 5 h. The WB analysis of endogenous levels of c-Fms, RANK, and Rab11b was done. β-actin was used as a loading control. The shown data was the representative of two independent repeats. (**E**, **F**) Cell viability was assessed by the cellular ATP content measurement using the Cell Titer Glo Assay system. After stimulated with RANKL (300 ng/mL) for 3 days, RAW-D cells expressing GFP or one of three different types of GFP-Rab11b was treated with CHX (20 μg/mL) and simultaneously with or without CLQ (10 μM) for 5 h (**E**), and with or without MG132 (10 μM) for 5 h (**F**). The values were the average of triplicate determinations with S.D indicated by error bars. n.s. nonsignificant (Student's *t*-test). Data were representative of two independent repeats.

Publisher's Note: MDPI stays neutral with regard to jurisdictional claims in published maps and institutional affiliations.

© 2020 by the author. Licensee MDPI, Basel, Switzerland. This article is an open access article distributed under the terms and conditions of the Creative Commons Attribution (CC BY) license (http://creativecommons.org/licenses/by/4.0/).
